# Characterization of PR-10 genes from eight Betula species and detection of Bet v 1 isoforms in birch pollen

**DOI:** 10.1186/1471-2229-9-24

**Published:** 2009-03-03

**Authors:** Martijn F Schenk, Jan HG Cordewener, Antoine HP America, Wendy PC van't Westende, Marinus JM Smulders, Luud JWJ Gilissen

**Affiliations:** 1 Plant Research International, Wageningen UR, Wageningen, the Netherlands; 2 Allergy Consortium Wageningen, Wageningen UR, Wageningen, the Netherlands

## Abstract

**Background:**

Bet v 1 is an important cause of hay fever in northern Europe. Bet v 1 isoforms from the European white birch *(Betula pendula) *have been investigated extensively, but the allergenic potency of other birch species is unknown. The presence of Bet v 1 and closely related PR-10 genes in the genome was established by amplification and sequencing of alleles from eight birch species that represent the four subgenera within the genus *Betula*. Q-TOF LC-MS^E ^was applied to identify which PR-10/Bet v 1 genes are actually expressed in pollen and to determine the relative abundances of individual isoforms in the pollen proteome.

**Results:**

All examined birch species contained several PR-10 genes. In total, 134 unique sequences were recovered. Sequences were attributed to different genes or pseudogenes that were, in turn, ordered into seven subfamilies. Five subfamilies were common to all birch species. Genes of two subfamilies were expressed in pollen, while each birch species expressed a mixture of isoforms with at least four different isoforms. Isoforms that were similar to isoforms with a high IgE-reactivity (Bet v 1a = PR-10.01A01) were abundant in all species except *B. lenta*, while the hypoallergenic isoform Bet v 1d (= PR-10.01B01) was only found in *B. pendula *and its closest relatives.

**Conclusion:**

Q-TOF LC-MS^E ^allows efficient screening of Bet v 1 isoforms by determining the presence and relative abundance of these isoforms in pollen. *B. pendula *contains a Bet v 1-mixture in which isoforms with a high and low IgE-reactivity are both abundant. With the possible exception of *B. lenta*, isoforms identical or very similar to those with a high IgE-reactivity were found in the pollen proteome of all examined birch species. Consequently, these species are also predicted to be allergenic with regard to Bet v 1 related allergies.

## Background

Birch trees grow in the temperate climate zone of the northern hemisphere and release large amounts of pollen during spring. This pollen is a major cause of Type I allergies. The main birch allergen in northern Europe is a pathogenesis-related class 10 (PR-10) protein from the European white birch *(Betula pendula) *termed Bet v 1 [1,2]. Pollen of other Fagales species contains PR-10 homologues that share epitopes with Bet v 1 [3], as do several fruits, nuts and vegetables [4-7]. An IgE-mediated cross-reaction to these food homologues causes the so-called oral allergy syndrome (OAS) [8,9]. PR-10 proteins constitute the largest group of aeroallergens and are among the four most common food allergens [10].

The genus *Betula *encompasses over 30 tree and shrub species that are found in diverse habitats in the boreal and temperate climate zone of the Northern Hemisphere. The taxonomy of the *Betula *genus is debated, as is the number of recognized species. The genus is either divided into three, four or five groups or subgenera [11-13]. *B. pendula *occurs in Europe and is the only species whose relation to birch pollen allergy has been extensively investigated. Sensitization to birch pollen is also reported across Asia and North America, where *B. pendula *is not present [14,15]. Other *Betula *species occur in these areas, but their allergenic potency is unknown. *Betula *species may vary in their allergenicity as variation in allergenicity has been found among cultivars of apple [16-18], peach and nectarine [19], and among olive trees [20].

PR-10 proteins are present as a multigene family in many higher plants, including Gymnosperms as well as Monocots and Dicots [21-23]. The classification as PR-proteins [24] is based on the induced expression in response to pathogen infections by viruses, bacteria or fungi [25-27], to wounding [28] or to abiotic stress [29,30]. Some members of the PR-10 gene family are constitutively expressed during plant development [31] or expressed in specific tissues [23]. Multiple PR-10 genes have been reported for *B. pendula *as well [32]. mRNAs of these genes have been detected in various birch tissues, including pollen [1,33,34], roots, leaves [28,30], and in cells that are grown in a liquid medium in the presence of microbial pathogens [27]. PR-10 genes share a high sequence similarity and form a homogeneous group. Homogeneity is believed to be maintained by concerted evolution [35]. Arrangements of *PR-10 *genes into clusters, such as found for Mal d 1 genes in apple, may facilitate concerted evolution [22].

Several Bet v 1 isoforms have been described for *B. pendula *[1,32-34,36], including both allergenic and hypoallergenic isoforms [37]. Individual *B. pendula *trees have the genetic background to produce a mixture of Bet v 1 isoforms with varying IgE-reactivity [32]. The relative abundance of individual isoforms at the protein level will influence the allergenicity of the pollen. Molecular masses and sequences of tryptic peptides from Bet v 1 can be determined by Q-TOF MS/MS [38]. The recently developed Q-TOF LC-MS^E ^method enables peptide identification, but has the additional advantage of being able to determine relative abundances of peptides in a single run [39]. By quantifying isoforms with a known IgE-reactivity [37], the allergenicity of particular birch trees can be predicted. The existence of allergenic and hypoallergenic isoforms indicates that PR-10 isoforms vary in allergenicity, and some PR-10 isoforms do not bind IgE at all. This has already been demonstrated for two truncated Bet v 1 isoforms [33]. Therefore, not all PR-10 isoforms are necessarily isoallergens.

Knowledge on the allergenicity of birch species may facilitate selection and breeding of hypoallergenic birch trees. To investigate the presence and abundance of Bet v 1 isoforms in *Betula *species that are potential crossing material, we: (*I*) cloned and sequenced *PR-10 *alleles from eight representative *Betula *species to detect *PR-10 *genes at the genomic level, (*II*) applied Q-TOF LC-MS^E ^to identify the pollen-expressed Bet v 1 genes, (*III*) determined relative abundances of isoforms in the pollen proteome, and (*IV*) compared these isoforms to isoforms with a known IgE-reactivity.

## Results

**Figure 1:**
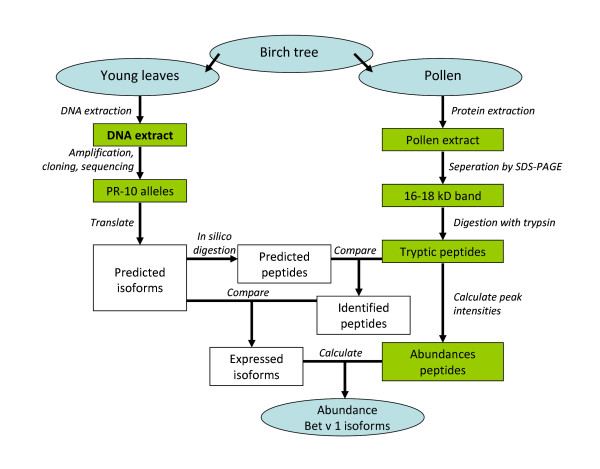
**Study workflow diagram**. This diagram gives an overview of the experimental steps (green boxes) and analyses (white boxes) performed in this study.

This study encompasses several experimental and analytical steps, involving both genomics and proteomics. All main steps have been summarized in Fig. 1.

### PR-10 subfamilies

**Figure 2:**
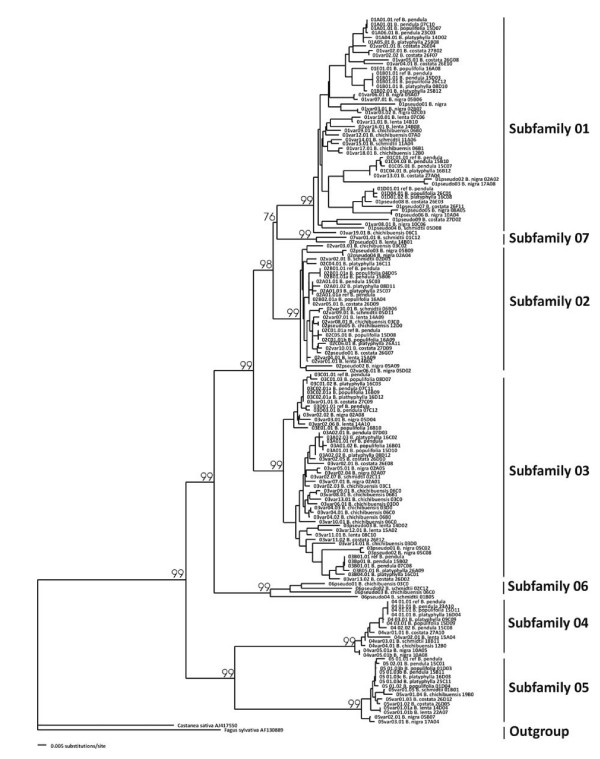
**Grouping of PR-10 sequences into subfamilies**. Clustering of the PR-10 sequences from eight *Betula *species in a Neighbor Joining tree with Kimura two-parameter distances. The sequences group into seven subfamilies. Bootstraps percentages on the branches indicate support for these groups.

We examined eight *Betula *species for the presence of PR-10 genes by sequencing 1029 individual clones in both directions (Table 1). Sequences that contained PCR artifacts were excluded by combining information from independent PCRs. The Open Reading Frames (ORF) of the sequences were highly conserved, making the alignment straightforward. The consensus sequence of the exon had 452 positions excluding the 31 bps in the primer regions. 228 out of the 274 variable consensus positions were phylogenetically informative. The sequences grouped into seven well-supported clusters in the Neighbor Joining (NJ) tree (Fig. 2). Five clusters coincided with the division between subfamilies as found in *B. pendula *[32]. Two new subfamilies (06 and 07) were identified, which occurred only in two species, contrary to the previously described subfamilies 01 to 05 that were found in all species (Table 1). In all sequences, an intron was located between the first and second nucleotide of codon 62. This intron was highly variable in length and composition, which was an additional characteristic for inferring the proper subfamily. Intron sequences were excluded from the phenetic/phylogenetic analysis because introns evolve at a different speed compared to exons.

**Table 1:** Number of identified PR-10 sequences in nine birch species.

Species	Number of sequenced clones	Subfamily 01		Subfamily 02		Subfamily 03		Subfamily 04		Subfamily 05		Subfamily 06		Subfamily 07		Total	
		Seqs	Genes	Seqs	Genes	Seqs	Genes	Seqs	Genes	Seqs	Genes	Seqs	Genes	Seqs	Genes	Seqs	Genes
**Subgenus *Betulaster:***																	
***B. nigra***	**155**	10	6	4	3	7	4	2	1	2	1	-	-	-	-	**25**	**15**
																	
**Subgenus *Neurobetula:***																	
***B. chichibuensis***	**170**	5	4	3	2	10	7	1	1	1	1	2	2	-	-	**22**	**17**
***B. schmidtii***	**184**	3	2	3	2	1	1	1	1	1	1	2	2	1	1	**12**	**10**
																	
**Subgenus *Betulenta:***																	
***B. lenta***	**106**	3	2	3	2	4	4	1	1	2	1	-	-	1	1	**14**	**11**
																	
**Subgenus *Betula:***																	
***B. costata***	**103**	9	8	3	2	5	5	1	1	2	1	-	-	-	-	**20**	**17**
***B. pendula***	**102**	5	3	2	2	5	4	2	1	2	1	-	-	-	-	**16**	**11**
***B. plathyphylla***	**103**	6	4	4	3	6	3	2	1	2	1	-	-	-	-	**20**	**12**
***B. populifolia***	**106**	4	4	4	2	5	3	2	1	2	1	-	-	-	-	**17**	**11**
B. pendula reference*^2^	**-**	-	4	-	3	-	4	-	1	-	1	-	-	-	-	**-**	**13**

The number of clones sequenced in both directions and the number of identified sequences and genes are shown per species.^1 ^Subfamily 01 to 05 were previously identified [32], while subfamily 06 and 07 are new. Homology to mRNA sequences suggests that subfamily 01 and 02 are expressed in pollen.

*1 Species were diploid (2n) as measured by flow cytometry. The identification of alleles of a single gene is based on the criterion of having > 98.5% sequence similarity, and by allowing maximally two alleles per gene.

*2 Genes identified in *B. pendula *[32].

### PR-10 sequences and genes

We recovered 12 to 25 unique PR-10 sequences per species, adding up to 146 sequences in total (Table 1). Out of the 134 unique sequences, over 100 sequences have never been described before. *B. pendula*, *B. plathyphylla *and *B. populifolia *are closely related members of the subgenus *Betula *and consequently had multiple alleles in common. These species shared one allele with *B. costata*, which is another member of the subgenus *Betula*. We applied a predefined cut-off level of 98.5% to attribute all sequences to different genes, while allowing maximally two alleles per gene per species. These criteria coincided in the majority of cases, but several genes of *B. chichibuensis *in the large cluster in subfamily 03 and of *B. lenta *in subfamily 02, and the genes 02A/02B and 03C/03D in *B. pendula *were more than 98.5% similar. Table 1 shows the total number of identified PR-10 genes per species. Out of the 13 genes that have previously been identified in *B. pendula *(Table 1; Fig. 2), 11 genes were recovered from the newly sequenced *B. pendula *cultivar 'Youngii'. This study identified no new genes in this cultivar. This indicates that the majority of genes has been recovered by sequencing over 100 clones per species, and that only a small number of genes might be missing in the dataset.

Homologues of the PR-10 genes of *B. pendula *were identified in *B. populifolia *and *B. plathyphylla*. Sequences from these species were labeled according to the procedure described by Gao *et al*. [22] that was previously used for *B. pendula *[32]. These labels consist of the subfamily's number, followed by a letter for each distinct gene, then a number for each unique protein variant and an additional number referring to silent mutations. When applicable, an additional letter indicates variations in the intron. The PR-10 genes in *B. costata *displayed a considerable degree of homology to the genes in *B. pendula*, but differentiating homologues and paralogues was not always possible. It was not possible to differentiate between homologues and paralogues of the PR-10 genes in *B. lenta*, *B chichibuensis*, *B. nigra*, and *B. schmidtii*. Rather than developing a separate denomination scheme for each species, we labeled sequences with the PR-10 subfamily number, followed by a number for each unique protein variant and an additional number referring to silent mutations. This facilitates the protein analysis which distinguishes protein variants rather than separate alleles or genes.

The PR-10 gene copy number varied between different birch species. This is caused by evolutionary processes such as duplication, extinction, and recombination. The overall clustering pattern appears to reflect a combination of such events. Genes from the same species tend to group close to each other on several positions in the NJ tree (Fig. 2). Examples are the clusters of highly similar sequences from *B. costata *in subfamily 01 and from *B. chichibuensis *in subfamily 03, which either reflect unequal crossing-over, gene conversion or duplication events. The *B. populifolia *genome harbors two clear examples of unequal crossing-over. Allele *01E01.01 *is a recombination between the *01A *gene and the *01B *gene. The first part matches exactly to allele *01A01.01*, while the second part differs by 1 SNP from *01B01.01 *with position 267 of the ORF as the point of recombination. Both original genes were also present. Similarly, allele *03E01.01 *is a recombination between the *03B *gene and the *03D *gene. In this case, the recombination probably occurred without gene duplication, since the original *03B *gene, as present in *B. pendula*, was absent.

### PR-10 protein predictions

Not all PR-10 alleles will be expressed as a full-sized protein. 112 unique sequences had an intact ORF, while the remaining 22 sequences contain early stop codons or indels in the ORF that result in frame shifts followed by an early stop codon. The latter sequences were denoted as pseudogenes, although it cannot be excluded that these sequences produce truncated proteins. We calculated K_a_/K_s _ratios within each subfamily. The suspected pseudogenes displayed higher K_a_/K_s _ratios than the alleles with an intact ORF in the subfamilies 01, 02 and 03 (Table 2). This points to an alleviated selection pressure in the pseudogenes. The other PR-10 subfamilies do not contain sufficient numbers of both genes and pseudogenes to perform this comparison. The majority of sequences had 5' splicing sites of AG:GT and 3' splicing sites of AG:GC, AG:GT or AG:GA, which is in concordance with known motifs for plant introns. Notable exceptions were: an AC:GT (*B. schmidtii*, 01pseudo04) and an AG:AT (*B. nigra*, 04var05.01a) 5' splicing site, an AC:GC (*B. schmidtii*, 01pseudo04) and a TG:GC (*B. nigra*, 02pseudo04) 3' splicing site, and two deletions (*B. costata*, 01pseudo05 and 02pseudo01) at the 3' end of the intron. Except for the AG:AT splicing site, all exceptions belonged to sequences that were denoted as pseudogenes, providing additional evidence for these designations.

**Table 2:** Sequence conservation within subfamilies of the PR-10 family among eight *Betula *species.

Subfamily	01	02	03	04	05	06	07
**Sequences with an intact ORF**							
*n *=	33	19	39	6	14	0	1
K_a_/K_s _ratio	0.18	0.27	0.10	0.36	0.09	*n. d*.	*n. d*.
Range substitutions	0 – 16	0 – 9	0 – 8	0 – 6	0 – 4	*n. d*.	*n. d*.
Average # substitutions	7.0	3.1	2.8	3.3	0.9	*n. d*.	*n. d*.
							
**Pseudogene sequences**							
*n *=	9	5	3	0	0	4	1
K_a_/K_s _ratio	0.38	0.30	0.20	*n. d*.	*n. d*.	0.57	*n. d*.

*n *= number of unique sequences. K_a_/K_s _ratio = ratio between non-synonymous and synonymous mutations. Range substitutions = minimum and maximum number of amino acid substitutions in pair wise comparisons between sequences of the same subfamilies. *n. d*. = not determined.

**Figure 3:**
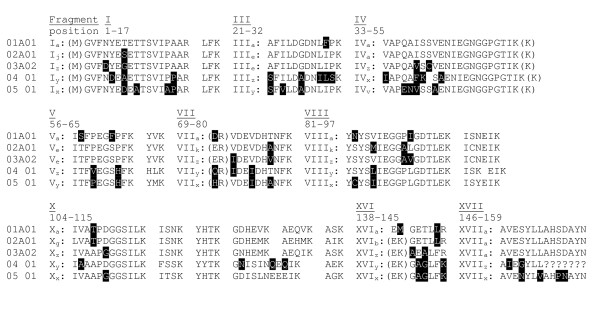
**Alignment of theoretical tryptic peptides of PR-10 proteins in *B. pendula *'Youngii'**. For clarity, one amino acid sequence is shown per subfamily. Only those fragments that are large enough to be detected by Q-TOF LC-MS/MS are labeled. Variable amino acids are marked in black.

Depending on the subfamily, K_a_/K_s _ratios ranged from 0.09 to 0.36 for sequences with an intact ORF (Table 2), indicating strong purifying selection. The PR-10 alleles in birch encode a putative protein that consists of 160 amino acids, yielding a relative molecular mass of approximately 17 kDa. The only exception is 01var17.01 in *B. chichibuensis*, which contains an indel that results in the deletion of two amino acids. The allelic variation is lower at the protein level than at the nucleic acid level, which is consistent with the low K_a_/K_s _ratios. Hence, the 112 unique genomic sequences encode 80 unique isoforms. The *PR-10.05 *gene is an extreme example for which only four putative isoforms are predicted, despite the presence of 14 allelic variants. One of these isoforms is predicted in all species except *B. nigra*. Parts of the PR-10 protein sequences are highly conserved, as is demonstrated in the amino-acid alignment of five PR-10 isoforms (one per subfamily) from *B. pendula *(Fig. 3). The most prominent region lies between Glu_42 _and Ile_56 _and contains only a single amino acid variation among all 80 isoforms. A phosphate-binding loop with the sequence motive GxGGxGx characterizes this region. Additional conserved Glycine residues are present at positions 88, 89, 92, 110 and 111.

### Bet v 1 expression in pollen

**Figure 4:**
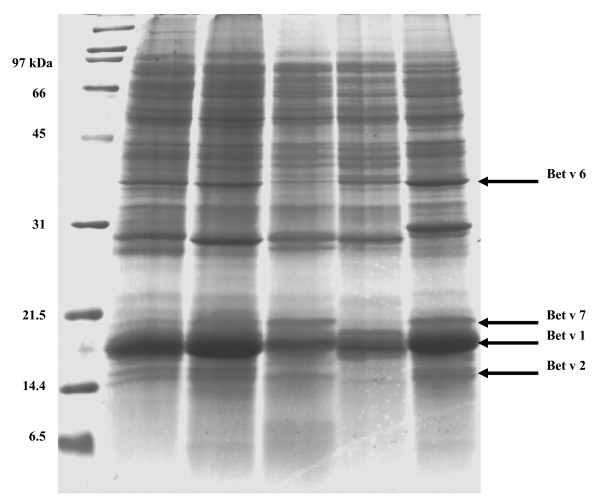
**SDS-PAGE analysis of birch pollen extracts**. (Lane 1) *B. chichibuensis*, (2) *B*. *costata*, (3) *B. nigra*, (4) *B. lenta *and (5) *B. pendula*. Bands of allergens that were analyzed and identified with Q-TOF LC-MS/MS are indicated by arrows. (M) LMW size marker proteins.

The presence of Bet v 1-like proteins was examined in pollen of *B. nigra*, *B. chichibuensis*, *B. lenta*, *B. costata *and *B. pendula *'Youngii'. Pollen proteins were solubilized in an aqueous buffer and analyzed by SDS-PAGE. Each sample displayed an intense protein band after CBB-staining at the expected molecular mass of Bet v 1, between 16–18 kDa (Fig. 4), while other intense bands were visible at 28 kDa and 35 kDa. No 16–18 kDa band was visible when the pellet that remained after extraction was separated by SDS-PAGE (not shown), indicating the efficiency of the extraction procedure with regard to Bet v 1.

To establish the identity of the proteins in the 16–18 kDa band, we cut out this band from the lane of *B. pendula *(Fig. 4) and performed in-gel digestion with trypsin. Q-TOF LC-MS/MS analysis of the tryptic peptides yielded multiple Bet v 1 isoforms (details given below). The bands just above and below the 16–18 kDa band were also sequenced and checked for the presence of Bet v 1. The lower band at 14 kDa contained birch profilin (Bet v 2; GenBank AAA16522; 2 peptides, coverage 24%) and contained no Bet v 1 fragments. The higher band at 19 kDa contained birch cyclophilin (Bet v 7; CAC841116; 3 peptides, coverage 28%) and some minor traces of Bet v 1 (Bet v 1a; CAA33887; 1 peptide, coverage 14%). Bollen *et al*. [4] detected a band of ~35 kDa when purified Bet v 1 was analyzed by SDS-PAGE, consisting of (dimeric) Bet v 1. We identified the intense band at ~35 kDa in our *B. pendula *extract as isoflavone reductase (Bet v 6; GenBank AAG22740; 19 peptides, coverage 49%) and detected no Bet v 1 fragments in this band.

### Analysis of Bet v 1 isoforms by Q-TOF LC-MS^E^

The tryptic digests of the 16–18 kDa bands were examined in detail to elucidate the expression of separate Bet v 1 isoforms in pollen. Trypsin cleaves proteins exclusively at the C-terminus of Arginine and Lysine. Fig. 3 shows an example of the fragments I to XVII that are theoretically formed after tryptic digestion of isoforms from the subfamilies 01 to 05. Isoforms of different subfamilies can be discriminated by several fragments on the basis of peptide mass and sequence. The number of discriminating fragments becomes lower for Bet v 1 isoforms within a subfamily. A new mass spectrometric technique called Q-TOF LC-MS^E ^allows simultaneous identification and quantification of peptides (see Method section for details). A distinct feature of the LC-MS^E ^procedure is that information is obtained for all peptides. This contrasts MS/MS, in which a subset of peptides is selected for fragmentation. A software program analyses the data, while using a search database for interpretation of the fragmentation spectra. This database contained the sequence information of all PR-10 isoforms described in this paper and of previously described PR-10 isoforms from *B. pendula *[32].

The LC-MS^E ^results indicated that PR-10 proteins of subfamily 01 and 02 are expressed in the pollen of the five examined birch species. We found no evidence for the expression of genes from subfamilies 03 to 07 in pollen. For example, we identified 22 Bet v 1 peptide fragments in *B. pendula *(Table 3), all of which were predicted from the gDNA sequences. Eight detected peptides could distinguish between isoforms of subfamily 01 and 02. The *B. pendula *genome contains seven genes from subfamily 01 and 02. The expression of four of these (*01A*, *01B*, *01C*, *02C*) was confirmed (Table 3). Sequence coverage of the expressed isoforms amounted to 71 to 79% (Table 3). Four peptides were specific for isoform 01B01, while one peptide was specific for isoform 02C01. Two peptides were specific for both isoforms of gene *01A*, while two others were specific for both isoforms of gene *01C*. Isoforms 02A01 and 02B01 could not be separated, so either one or both of them are expressed. Table 3 also shows the peptide fragments that were long enough to be detected in the tryptic digest, but were not observed. Information on absent fragments can be used to exclude expression of particular isoforms, such as isoform 01D01 in *B. pendula*.

**Table 3:** Peptides fragments of PR-10 isoforms in pollen from five *Betula *species as identified by Q-TOF LC-MS^E^.

Species	Fragment		I	III	IV	V	VII	VIII	X	XVI	XVII	Sequence coverage
	Isoform	Gene							*^2^			
*B. pendula*	01A01	1A	**A**	***a***	a	***a***	***a***	**A**	a?	***a***	a	79%
	01A06	1A	**A**	***a***	a	***b***	***a***	**A**	a?	***a***	a	79%
	01B01	1B	**B**	**B**	a	***b***	***a***	**C**	**B**	***a***	a	79%
	01C04	1C	**D**	***a***	a	***a***	***a***	**D**	***c?***	***a***	a	71%
	01C05	1C	**D**	***a***	a	***a***	***a***	**D**	***c?***	***a***	a	71%
	01D01	1D	**(E)**	***a***	a	**(C)**	**(C)**	**(E)**	a?	***a***	a	-
	
	02A01	2A	***j***	e	a	e	***k***	***k***	***g?***	(B*^3^)	a	74%
	02B01	2B	***j***	e	a	e	***k***	***k***	***g?***	(c*^3^)	a	74%
	02C01	2C	***j***	***F***	a	e	***k***	***k***	a?	(c*^3^)	a	74%
	
	03 *^1^		**(C), (*z*)**	e	**(*z*), (Y)**	e	**(Z), (*y*)**	**(*z*), (Y)**	**(z), (Y)**	**(*z*)**	a	-
	
	04 *^1^		**(Y)**	**(Z)**	**(X), (W)**	**(Z)**	**(X)**	**(X)**	**(X)**	**(Y)**	**(Z), (Y)**	-
	
	05 *^1^		**(X)**	**(Y)**	**(V)**	**(Y)**	**(W)**	**(W)**	**(W)**	**(X)**	**(X)**	-

*B. chichibuensis*	01var09	1A	***a***	***a***	a	***a***	***a***	***d***	a?	***a***	a	79%
	01var12	1B	**C**	***a***	a	***a***	***a***	***d***	a?	***a***	a	79%
	01var17	1C	***a***	***a***	a	***a***	**H**	**(H)**	***c?***	***a***	a	-
	01var18	1C	***a***	***a***	a	***a***	**H**	***d***	***c?***	***a***	a	71%
	01var19	1D	**(E)**	***a***	a	**(C)**	**(J)**	**(E)**	a?	***a***	a	-
	
	02var03	2A	***j***	e	**B**	e	***k***	***k***	a?	(c*^3^)	a	74%
	02var08	2B	***j***	**F**	a	e	***k***	***k***	a?	(c*^3^)	a	74%

*B. costata*	01var01	1A	**F**	***a***	a	***a***	***a***	***c***	a	***a***	a	79%
	01var02	1B	***a***	***a***	a	***a***	***a***	***c***	a	***a***	a	79%
	01var04	1C	***a***	**(C)**	a	***b***	**(E)**	***c***	**(D)**	***a***	a	-
	01var05	1D	***a***	***a***	a	***b***	***a***	***c***	**(E)**	***a***	a	71%
	01var13	1E	**D**	***a***	a	***a***	***a***	**D**	a	***a***	a	79%
	Unknown		**E**									?
	
	02var05	2A	***j***	**(J)**	a	e	***k***	***k***	a	(c*^3^)	a	-
	02var10	2B	***j***	**G**	a	e	***k***	***k***	a	(c*^3^)	a	74%
	Unknown		**F**									?

*B. lenta*	01var10	1A	**G**	**A**	a	**(A)**	**(F)**	***d***	a	(a*^3^)	a	60%
	01var11	1A	**G**	**A**	a	**(A)**	**(G)**	***d***	a	(a*^3^)	a	60%
	01var16	1B	**B**	**D**	a	**D**	**A**	***d***	a	(a*^3^)	a	74%
	
	02var01	2A	***j***	e	a	e	***k***	***k***	a	(c*^3^)	a	74%
	02var04	2B	***j***	**H**	a	e	***k***	***k***	a	(c*^3^)	a	74%
	02var07	2C	***j***	**F**	a	e	***k***	***k***	a	(c*^3^)	a	74%

*B. nigra*	01var03	1A	**B**	***a***	a	**B**	***a***	**F**	a	***a***	a	79%
	01var06	1B	***a***	***a***	a	***a***	-	**C**	a	***a***	a	74%
	01var07	1C	***a***	***a***	a	***a***	***a***	**C**	a	***a***	a	79%
	01var08	1D	***a***	***a***	a	***a***	***a***	**D**	**F**	***a***	a	79%
	
	02var06	2A	**J**	**K**	a	e	**K**	**K**	a	(c*^3^)	a	74%
	unknown			**F**								?

Each isoform is displayed on a separate line. When isoforms are encoded by the same gene this is indicated in the third column. Note that gene labels in one species do not correspond to gene labels in other species. Peptide fragments are shown at the top of the table and are labelled with Roman numbers as indicated in Fig. 3. Each variant of these fragments is displayed in the Table by a letter. Bold capital letters indicate that a fragment is unique for the isoforms of a particular gene. Bold italic letters indicate that a fragment is unique for the isoforms of a particular subfamily. Letters displayed between brackets indicate that a particular fragment was predicted, but was **absent **in the PR-10 mixture. Finally, the last column displays the coverage of the total protein sequence, including the fragments that were too small to be detected (II, VI, IX, XI, XII, XIII, XIV, XV). Fig. 3 displays the representative amino acid sequences of the isoforms 01A01 and 02A01.

*1 The isoforms in subfamily 03 to 05 were summarized into a single row and not displayed for the other species, because specific peptides were not detected in any of the species.

*2 Fragments X_a _and X_g _have exactly the same mass and cannot be distinguished. The peak of peptide X_c _overlaps with the first isotope peak of peptide X_a = g _because they differ exactly 1 Da in size and have the same charge. As a consequence, X_c _cannot be identified separately.

*3 The XVI-peptides are not always detected because of their small size.

Altogether, at least 4 to 6 isoforms were expressed in each of the five examined species. In total, the presence of unique peptides confirmed the expression of 14 isoforms among the five species in total (Table 3). An additional 15 isoforms lacked one or more unique peptides to distinguish them from other isoforms or from each other, but several of these must be expressed. The expression of five isoforms was ruled out, because multiple unique peptides from these variants were lacking from the peptide mixture. Two identified peptides in *B. costata *and one peptide from *B. nigra *did not match to any sequence that was recovered from these species. These peptides belong to "unknown isoforms" (Table 3) and this indicates that the sequences that encode these isoforms are missing from the dataset. Finally, conflicting evidence was found for expression of the isoforms 01var10 and 01var11 in *B. lenta*. Two peptides that were unique for these isoforms were detected, while three peptides that were expected if the isoforms would be expressed were lacking. Expression of an allele that is missing from our dataset is a more likely explanation than the expression of 01var10 or 01var11.

### Quantification by Q-TOF LC-MS^E^

We determined the relative amounts of individual Bet v 1 isoforms in pollen from *B. pendula *'Youngii' (Table 4). This information can be deduced from the peak intensities of Bet v 1 peptides in the tryptic digest. Not all identified fragments can be used for quantification, because the peak detection algorithm groups peaks with highly similar masses and retention times together, also when they might belong to different fragments. For example, fragment I_a _(1854,91 Da) and VII_a _(1854,89 Da) have a retention time that is marginally different, causing a strong overlap in peak area. The relative amounts of two isoforms could be estimated directly: peptide III_f _is unique for isoform 02C01 and comprises 17% of all fragment III-variants, while peptides III_b _and X_b _are unique for 01B01 and comprise 18–19% of all fragment III and X-variants. The isoforms 02A01 and 02B01 could not be separated, but together they comprise 13% of the mixture based on fragment III_e_. The relative amounts of the other isoforms were estimated indirectly. Isoform 01A06 and 01B01 share fragment V_b_, which comprises 23% of all fragment V-variants. 01A06 is thus estimated to comprise 4–5% of the mixture. The ratio between 01B01 and 01C04 plus 01C05 can be deduced from fragment I_b_. 01C04 plus 01C05 are thus estimated to comprise 6% of the mixture. This leaves 40–41% of the total amount of Bet v 1 for isoform 01A01.

**Table 4:** Quantification of identified peptides by Q-TOF LC-MS^E ^in the pollen of *B. pendula *'Youngii'.

Fragment		I*^1^	III	IV	V	VII	VIII*^1^	X *^2^	XVII	Direct coverage estimate	Indirect coverage estimate	Subfamily	Direct estimate
**Isoform**	**Gene**												
**01A01**	**1A**	Ia: n.q.	IIIa: 51	IVa: 100	Va: 46	VIIa: 75	VIIIa: n.q.	Xa+g+c: 82	XVIIa: 100	-	4–41%	**01**	68–75%
**01A06**	**1A**	Ia: n.q.	IIIa: 51	IVa: 100	Vb: 23	VIIa: 75	VIIIa: n.q.	Xa+g+c: 82	XVIIa: 100	-	4–5%		
**01B01**	**1B**	Ib: 69	IIIb: 19	IVa: 100	Vb: 23	VIIa: 75	VIIIc: n.q.	Xb: 18	XVIIa: 100	18–19%	-		
**01C04/01C05**	**1C**	Id: 31	IIIa: 51	IVa: 100	Va: 46	VIIa: 75	VIIId: 100	Xa+g+c: 82	XVIIa: 100	-	6%		
**01D01**	**1D**	**Ie: 0**	IIIa: 51	IVa: 100	Vc: 0	VIIc: 0	VIIIe: 0	Xa+g+c: 82:	XVIIa: 100	0%	-		

**02A01/02B01**	**2A**	Ia: n.q.	IIIe: 13	IVa: 100	Ve: 32	VIIk: 25	VIIIk: n.q.	Xa+g+c: 82	XVIIa: 100	13%	-	**02**	25–32%
**02C01**	**2C**	Ia: n.q.	IIIf: 17	IVa: 100	Ve: 32	VIIk: 25	VIIIk: n.q.	Xa+g+c: 82	XVIIa: 100	17%	-		

Numbers indicate the relative amount of fragment variants compared to the total amount of homologues fragments. Amounts were averaged over the two duplicates. Note that quantification was not possible for all peptide variants^1,2 ^and that the displayed abundances indicate the relative amounts among those variants that could be quantified. n.q. = not possible to quantify.

* 1 Quantification was not possible for all the peptide variants, because I_a _(1854,91 Da) and VIII_a _(1854,89 Da), and I_j _(1840,89 Da) and VIII_c _(1840,88 Da) had a similar mass. Fragment VIII_k _overlaps with a keratin peptide.

* 2 Fragments X_a _and X_g _have exactly the same mass and cannot be distinguished. The peptide peak of X_c _overlaps with the first isotope peak of peptide X_a = g _because they differ exactly 1Da in size and have the same charge. X_c _cannot be identified as a result.

Isoform 01A01 is identical to isoform Bet v 1a, which had the highest IgE-reactivity in several tests performed by Ferreira *et al*. [37]. Pollen of *B. costata*, *B. nigra *and *B. chichibuensis *contained isoforms that are highly similar to Bet v 1a and differ by only 1–3 amino acids from this isoform. We determined the expression of individual Bet v 1 isoforms in a similar fashion as reported for *B. pendula*. The Bet v 1a-like isoforms were estimated to comprise 38% (*B. chichibuensis*), 36–44% (*B. nigra*) and 36–41% (*B. costata*) of the total amount of Bet v 1. *B. lenta *differed from the other species, because the isoform with the highest similarity to Bet v 1a differed by seven amino acids. This isoform was estimated to comprise 12–19% of the total amount of Bet v 1. The expression of subfamily 01 isoforms relative to subfamily 02 isoforms was another major difference between *B. lenta *and the other species. In *B. lenta*, subfamily 02 accounted for 74–83% of the total amount of Bet v 1, compared to 25–40% in *B. pendula*, *B. nigra *and *B. chichibuensis *and 49–56% in *B. costata*.

## Discussion

### PR-10 gene family organization and evolution

The presence and diversity of Bet v 1 and closely related PR-10 genes in eight birch species was established by amplification and sequencing of more than 100 clones per species. The eight species belong to four different subgenera/groups in the genus *Betula *[13] and thereby represent a large part of the existing variation within the genus. Each birch species contains PR-10 genes, as could be expected given the broad range of plant species in which PR-10 genes are found [21-23]. The PR-10 genes grouped into subfamilies, as previously reported for *B. pendula *[32]. Five subfamilies were recovered from all species. Two new subfamilies were identified, but these were each restricted to two species and were mostly composed of pseudogenes.

The PR-10 subfamily has a complex genomic organization. Differentiating between paralogues and homologues was not possible beyond closely related species. One likely explanation is concerted evolution, for which cladistic evidence was found (Fig. 2). Concerted evolution causes genes to evolve as a single unit whose members (occasionally) exchange genetic information through gene conversion or unequal crossing-over [40]. Tandemly arranged genes have increased conversion rates, while such an arrangement is a prerequisite for the occurrence of unequal crossing-over [41]. Most *PR-10 *genes in apple are arranged in a duplicated cluster [22], thus facilitating the main mechanisms for concerted evolution. We obtained two alleles that appear the direct result of unequal crossing-over between Bet v 1 genes. On a higher taxonomic level, cladistic evidence for concerted evolution is present in the overall gene tree of the PR-10 family [35], as sequence divergence is generally smaller between different genes from the same species than between genes from different species.

Nei and Rooney [42] suggested that a combination of recent gene duplications and purifying selection could also explain why tandem gene duplicates appear similar. In their model of birth-and-death evolution of genes, new genes arise due to gene duplications, evolve independently while undergoing purifying selection, and go extinct after becoming non-functional. Pseudogenes are characteristic for this process. The low K_a_/K_s _ratios clearly point to the occurrence of purifying selection. Pseudogenes are a common feature among the PR-10 genes in birch, since we recovered them from six out of eight species. As much as one-third of the recovered alleles in *B. nigra *had an interrupted ORF. We did not determine the potential expression of these alleles, since truncated isoforms would have migrated outside the 16–18 kDa band in the SDS-PAGE. None were, however, detected in the 14 kDa band. Basically, all ingredients for the "birth-and-death" model are present, except that independent evolution is questionable due to the presences of duplicates that resulted from unequal crossing-over. Moreover, the clustering of for example the *B. chichibuensis *alleles (Fig. 2) would suggest an extremely high number of recent duplications. Both processes of "birth-and-death" and concerted evolution may, therefore, be active in the PR-10 gene family. Regardless of the evolutionary processes, its outcome is clear: PR-10 proteins are homogenous as a group and even stronger so within subfamilies. The high homogeneity allowed us to use Q-TOF LC-MS^E ^to quantify the relative expression of separate Bet v 1 isoforms, because large differences in amino acid composition would have distorted the quantification.

### Bet v 1 expression

Which PR-10 genes are actually expressed in pollen and are thereby the true Bet v 1 allergens? We used Q-TOF analysis to investigate the expression of Bet v 1 isoforms in pollen of five *Betula *species. Isoforms from subfamily 01 and 02 were identified in birch pollen, confirming predictions based on mRNA expression [1,33,34]. The single gene in subfamily 05 that was present in all eight birch species, is homologous to *ãpr10c*, which has a high basal transcription level in roots and a relatively lower basal transcription level in leaves [27,28,43]. Its expression is induced by copper stress [30] and during senescence in leaves [44]. Regarding subfamily 03, the genes PR-10.03C and 03D (= *γpr10a *and *γpr10b*) in *B. pendula *become transcriptionally upregulated upon infection of the leaves with fungal pathogens [27]. Their transcription is induced by wounding or auxin treatment in roots [28,43]. No data have been reported about the expression of the sequenced PR-10 genes in subfamilies 04, 06 and 07.

The pollen-expressed Bet v 1 genes are transcribed during the late stages of anther development [45], but which factors induce transcription is unknown. Bet v 1 is an abundant pollen protein that has been estimated to encompass 10% of the total protein in *B. pendula *pollen [46]. The Bet v 1 band was the most intense band in the SDS-PAGE gels of birch pollen extracts. Its exact abundance is difficult to estimate due to differences in extraction efficiency between different proteins. However, given the low amount of residual protein in the pellet, our results suggest that the abundance of Bet v 1 is higher than 10% of the total protein content and is likely to exceed 20%. The occurrence of Bet v 1 isoforms in *B. pendula *has previously been studied in a mixture of pollen from different trees by Swoboda *et al*. [34]. They analyzed tryptic digests of purified Bet v 1 isoforms by Plasma Desorption Mass Spectrometry (PDMS), a technique that only reveals peptide masses. We examined pollen from individual trees and analyzed the tryptic digests by Q-TOF LC-MS^E^, which reveals total masses of peptides and the underlying amino acid sequences, based on available sequence information. The ability to determine the peptide sequences yields more accurate information on expression of individual isoforms. We demonstrated that at least 4 to 6 isoforms were expressed in the pollen of one single tree of the birch species *B. pendula*, *B. nigra*, *B. chichibuensis*, *B. lenta *and *B. costata*. The actual number is likely to be higher since we could not discriminate each individual isoform due to the high similarity between some isoforms.

Q-TOF LC-MS^E ^has the advantageous ability to simultaneously separate, identify and quantify peptide fragments. A similar strategy has recently been followed by Chassaigne *et al*. [47]. They identified five peanut-specific peptide ions that were used as specific tags for the peanut allergenic proteins Ara h 1, Ara h 2, and Ara h 3. The relative intensity of the specific peptides even provided information on the processing history of the peanut material. Napoli *et al*. [48] also used mass spectrometry to analyze an Ole e 1 mixture of multiple isoforms and their post-translational modifications, which could not be separate completely by 2-Dimension gel electrophoresis. A disadvantage of using Q-TOF LC-MS^E ^instead of Q-TOF LC-MSMS in combination with 2D gel electrophoresis and Western blotting – in which allergic sera and specific anti-IgE antibodies are employed – is that our method does not distinguish IgE-binding isoforms from non-IgE-binding isoforms. Therefore, not all described PR-10 isoforms are necessarily true isoallergens.

We included no purification step in the extraction procedure apart from protein separation on SDS-PAGE. This minimizes the chance that certain isoforms are lost during purification, but the Bet v 1 protein band might be contaminated with other pollen proteins with a similar mass. Three peptides of the pollen allergen Bet v 7 were detected in the 16–18 kDa band, but the amount of Bet v 7 was estimated to be less than 2% of the amount of Bet v 1, based on the peak intensities of these peptides. All peptides with high peak intensities could be attributed to Bet v 1 isoforms. Full sequence coverage of Bet v 1 isoforms cannot be achieved by using only trypsin as a protease, as smaller peptides will be lost during peptide extraction from the SDS-PAGE gel. Proteases that cleave at other sites will yield peptides that cover part of the missing protein sequence. Coverage with Q-TOF LC-MS^E ^was 71–79% for the *B. pendula *isoforms, which is higher than the 57–60% coverage reported for Q-TOF MS/MS [38].

Swoboda *et al*. [34] estimated that, based on PDMS peak areas of peptides, the relative amount of Bet v 1a (= PR-10.01A01) in the pollen mixture from several *B. pendula *trees was at least 50% of the total amount of Bet v 1. Ferreira *et al*. [37] estimated the relative amounts of different Bet v 1 isoforms by NH_2_-terminal sequencing of purified natural Bet v 1 and reported a ~2:2:1 ratio for isoforms that respectively contain Ser, Thr and Ile at the 7^th ^amino acid position. This would correspond to expression of the isoforms 02A01+02B01+02C01: 01A01+01A06: 01B01+01C04+01C05 in pollen of *B. pendula *'Youngii'. When we sum our results in this way, a ratio of 30%:45%:25% is obtained. The similarity between our results and previously obtained estimates suggests that the quantities obtained from *B. pendula *'Youngii' are also representative for other *B. pendula *trees.

### Allergenicity

Ferreira *et al*. [37] distinguished Bet v 1 isoforms with a low, intermediate and high IgE-reactivity. Expression of the isoforms 01B01 (= Bet v 1d, low IgE-reactivity), 02C01 (= Bet v 1c, intermediate IgE-reactivity), 01C04 (= Bet v 1f, intermediate IgE-reactivity) and 01A01 (= Bet v 1a, high IgE-reactivity) in the pollen of *B. pendula *'Youngii' was confirmed by identification of unique peptides (Table 3). Isoforms of all three levels of IgE-reactivity were abundant and encompassed 35–38% (high), 22–24% (intermediate) and 18–19% (low) of the total amount of Bet v 1. This leaves 17–22% of the total Bet v 1 for isoforms with an unknown IgE-reactivity. We observed similar quantities in two other *B. pendula *cultivars as well (results not shown). Since *B. pendula *is known to be highly allergenic, the presence of isoforms with a high IgE-reactivity is apparently of determining influence on its allergenicity. Interestingly, people that are not yet sensitized to *B. pendula *pollen come into contact with several abundant isoforms. However, the ability of these isoforms to provoke an IgE-mediated response varies in patients that have become sensitized [49,50]. The factors that cause one isoform to develop into having a high IgE-reactivity and another isoform into having a low IgE-reactivity are currently unknown. The abundance of the isoforms may play a role, but as isoforms with intermediate or low IgE-reactivity are also present in considerable quantities, this is unlikely to be the only factor. Recently, Gao et al. [18], investigated the association of allelic diversity of Mal d 1 and allergenicity in ten pedigree-linked apple cultivars, and found that qualitative as well as quantitative factors were involved.

The opportunities for identifying birch trees that only express hypoallergenic isoforms are limited. The isoforms Bet v 1l and Bet v1d (= 01B01) are currently known as hypoallergenic [37]. The crystal-structure of Bet v 1l has been determined [51] and its allergenicity has recently been tested on a large group of patients [50]. However, none of the examined species contained Bet v 1l, despite thorough examination, and Bet v 1l may represent a sequencing artifact or an unexpressed allele. Only *B. pendula*, *B. populifolia *and *B. plathyphylla *contained 01B01. The most similar isoforms in the other species differed by at least five amino acids. In contrast, the highly allergenic isoform 01A01 is expressed in pollen of *B. pendula *'Youngii', while the *B. populifolia *genome also contains the 01A01 sequence. The other *Betula *species do not harbor Bet v 1a, but *B. chichibuensis*, *B. costata *and *B. nigra *contain isoforms that differ only by 1–3 amino acids from Bet v 1a. A high similarity between isoforms increases the chance that they share epitopes, although a few amino acid substitutions may influence the allergenicity drastically [52-54]. In all species, these isoforms are abundant. *B. lenta *forms an exception as the isoform most similar to Bet v 1a has a sequence similarity of 95.5% and encodes a protein that differs by seven amino acids.

## Conclusion

We identified 12 to 25 unique PR-10 sequences in each of eight different birch species. Application of Q-TOF LC-MS^E ^revealed that genes from two large subfamilies (01 and 02) were expressed in birch pollen. We showed that Q-TOF LC-MS^E ^allowed fast screening of Bet v 1 isoforms in birch pollen by determining presence and relative abundances of individual isoforms. The pollen of four birch species contained a mixture of Bet v 1 isoforms, with abundant levels of isoforms that were similar to isoforms with a high IgE-reactivity. We predict that the allergenic potency of these species will be high. *B. lenta *(subgenus *Betulenta*) lacked isoforms with a high similarity to isoforms with a high IgE-reactivity. This species and related species represent the most promising candidates for further screening of hypoallergenicity by for example skin prick tests or nasal challenges.

## Methods

### Plant Material

We collected young leaves from eight *Betula *species (Table 1). A recent phylogenetic analysis identified four groups (subgenera) of species within the genus *Betula *[13]. Each subgenus is represented by at least one species. Four species from the subgenus *Betula *were included here to cover the variation within this large group. Plant material was collected from the botanical collections of PPO Boskoop (Boskoop, the Netherlands), the Botanical Garden of Wageningen (Wageningen, the Netherlands) and the Von Gimborn Arboretum (Doorn, the Netherlands). Fresh leaf samples were analyzed by flow cytometry (Plant Cytometry Services, Schijndel, The Netherlands) to estimate the ploidy level. Diploid *(B. pendula) *and tetraploid *(B. pubescens) *controls were included. All examined accessions were diploid, thus keeping the number of expected sequences per accession small. During the flowering period of birch in April-May 2004, we collected pollen from the same trees for the species *B. nigra*, *B. chichibuensis*, *B. lenta*, *B. costata *and *B. pendula*.

### PCR, cloning and sequencing

DNA was extracted using the DNeasy Plant Mini kit (Qiagen) according to the manufacturer's instructions. *PR-10 *alleles were amplified from genomic DNA with two primer pairs that had been tested and used in previous research on *B. pendula *[32]. PCR amplification with both primer pairs was performed in 20 μl reactions containing 0.1 mM dNTP, PCR Reaction buffer (Eurogentec), 1.5 mM MgCl_2_, 0.6 μM forward primer, 0.6 μM reverse primer, 0.5 U *Taq *polymerase (Goldstar), and 20–80 ng template DNA. PCR reactions started with a heating step at 95°C for 15 minutes, followed by 16–24 cycles of denaturation at 94°C for 30 s, annealing at 50°C for 45 s, and extension at 72°C for 2 min. A final extension step of 10 min at 72°C was added after the last cycle. To reduce the number of PCR recombination artifacts, we used as few PCR cycles as possible. The minimum number of cycles required to generate sufficient product for cloning was assessed by visual inspection of the amplified products on agarose gel.

PCR products were purified with the MinElute PCR Purification Kit (Qiagen). Purified samples were ligated into the pGEM-T easy Vector (Promega) and established in *Escherichia coli *Subcloning Efficiency DH5α cells (Invitrogen) according to the manufacturer's instructions. White colonies were picked from agar plates and grown overnight at 37°C in freeze medium. We performed PCR-based screening with vector-specific M13 primers. These PCR products were purified with Sephadex G-50 (Millipore). The DYEnamic™ ET Terminator Cycle Sequencing Kit (Amersham) was used for the sequence reactions. We analyzed sequence products on a 96-capillary system (ABI 3730 × l). The genomic *Betula *sequences have been submitted to GenBank as EU526132–EU526277.

### Phenetic/phylogenetic analysis

Potential PCR artifacts (strand switching and base misincorporation) were excluded by retaining only those sequences that were confirmed in independent PCRs. We included one reference sequence per *B. pendula *gene in the dataset for comparison with previous results [32]. Nucleotide sequences were aligned using CLUSTAL W with a gap penalty of 10 and a gap extension penalty of 2. We excluded primer traces and introns from further analysis. A Neighbor Joining (NJ) tree was constructed with Kimura two-parameter distances. Gaps were treated as missing characters. Bootstrapping was carried out with 1,000 replicates in PAUP 4.0b10 [55]. The outgroup was composed of *PR-10 *sequences from *Castanea sativa *(AJ417550) and *Fagus sylvatica *(AJ130889), which are two related Fagales species. Parsimony analysis was conducted in PAUP as a heuristic search, while using the following options: 100,000 random additions while holding one tree at each step, TBR branch swapping, the MulTrees option switched on, and ACCTRAN for character optimization. A strict consensus tree was calculated for all of the most parsimonious trees. Branch support was assessed by bootstrap analysis comprising 10,000 replicates consisting of 10 random addition sequences with TBR branch swapping. Both analyses produced highly similar results; therefore, only the NJ analysis is shown.

### Protein search database

Nucleotide sequences were aligned codon-by-codon. We analyzed general selection patterns at the molecular level using DnaSp 4.00 [56]. The number of non-synonymous (K_a_) and synonymous substitutions (K_s_) per site were calculated from pair wise comparisons with incorporation of the Jukes-Cantor correction. Nucleotide data were translated. A Fasta database with the resulting protein sequences was used as a search database in the Q-TOF LC-MS^E ^analysis. As sequence information for the primer region was unavailable, we used the GenBank sequences X15877 (subfamily 01), X77265 (02), X77600 (03), and X77601 (05) to fill these gaps in sequences from the respective subfamilies. The initiating Methionine is removed during PR-10 protein synthesis [30,37] and was therefore removed from the predicted proteins. Protein sequences of birch PR-10 isoforms in GenBank (overview in: Schenk et al., 2006), keratin, trypsin and Bet v 7 (AJ311666) were added to the database.

### Protein extraction

Fifty mg of pollen were suspended in 1 ml of 0.05 M Tris-HCl (pH 7.5) following Cadot *et al*. [57], who found that yield and diversity of the extracted allergens are optimal at pH 7.5 for birch pollen. After incubation under constant shaking at room temperature for 1 hr, the pollen extract was centrifuged at 10.000 rpm for 5 min. The pellet was ground with an Eppendorf-fitting pestle. The extract was then shaken for another hour. The supernatant was collected after centrifugation (10.000 rpm; 5 min) and freeze dried for storage.

### SDS-PAGE

The freeze-dried protein extract was redissolved in 0.05 M Tris-HCL (pH 7.5) and analyzed with SDS-PAGE to localize Bet v 1-type proteins. Proteins were separated on a 15% w/v acrylamide SDS-PAGE gel with a 5% w/v stacking gel using the Mini-Protean II gel system (Bio-Rad). After staining with Coomassie BB R-250, the gels were scanned and analyzed by Quantity One (Bio-Rad) scanner software. Relative molecular masses were determined with SDS-PAGE Standards broad range markers (Bio-Rad).

The protein bands at a relative molecular mass of 16–18 kDa were cut out of the SDS-PAGE gel and processed essentially according to Shevchenko [58]. Bands were sliced into 1 mm^3^-pieces. Bands at 14, 19 and 35 kDa were cut out and analyzed as well. Proteins were reduced with DTT and alkylated with iodoacetamide. Gel pieces were dried under vacuum, and swollen in 0.1 M NaHCO_3 _that contained sequence-grade porcine trypsin (10 ng/μl, Promega). After digestion at 37°C overnight, peptides were extracted from the gel with 50% v/v acetonitrile, 5% v/v formic acid and dried under vacuum.

### Q-TOF LC-MS/MS and Q-TOF LC-MS^E^

Tryptic digests were analyzed by one-dimensional LC-MS in high-throughput configuration using the Ettan™ MDLC system (GE Healthcare), which was directly connected to a Q-TOF-2 Mass Spectrometer (Waters Corporation, UK). Samples (5 μl) were loaded on 5 mm × 300 μm ID Zorbax™ 300 SB C18 trap columns (Agilent Technologies), and peptides were separated on 100 μm i.d. × 15 cm Chromolith CapRod monolithic C18 capillary columns (Merck) at a flow rate of approximately 1 μl/min. A gradient was applied using two solvents. Solvent A contained an aqueous 0.1% formic acid solution and solvent B contained 84% acetonitrile in 0.1% formic acid. The gradient consisted of isocratic conditions at 5% B for 10 min, a linear gradient to 30% B over 40 min, a linear gradient to 100% B over 10 min, and then a linear gradient back to 5% B over 5 min. MS analyses were performed in positive mode using ESI with a NanoLockSpray source. As lock mass, [Glu^1^]fibrinopeptide B (1 pmol/μl) (Sigma) was delivered from the syringe pump (Harvard Apparatus, USA) to the reference sprayer of the NanoLockSpray source at a flow rate of 1 μl/min. The lock mass channel was sampled every 10 s.

To identify the 14, 16–18, 19 and 35 kDa bands, the Q-TOF-2 was operating in MS/MS mode for data dependent acquisition. The mass spectrometer was programmed to determine charge states of the eluting peptides, and to switch from MS to MS/MS mode for z ≥ 2 at the appropriate collision energy for Argon gas-mediated CID. Each resulting MS/MS spectrum contained sequence information on a single peptide. Processing and database searching of the MS/MS data set was performed using ProteinLynx Global SERVER (PLGS) v2.3 (Waters Corporation) and the NCBI non-redundant protein database, while taking fixed (carbamidomethylation) and variable (oxidation of Methionine) modifications into account.

After the identification of multiple Bet v 1 isoforms in the 16–18 kDa band, we analyzed the tryptic digest of this band with Q-TOF LC-MS^E^. The Q-TOF-2 was programmed to alternate between low and elevated levels of collision energy. Collision energy was 5 eV in MS mode and was increased in two steps from 28 to 40 eV in MS^E ^mode. Measuring time in both modes was 0.9 s with an interscan delay of 0.1 s. Unfragmented precursors predominate in low energy mode, while fragmented ions of the precursors are observed in high energy mode. Digests were analyzed in duplicate. MS^E ^data were analyzed according to the procedure described by Silva *et al*. [39] with the Expression module in PLGS. Different peptide components were detected with an ion detection algorithm, and then clustered by mass and retention time, followed by normalization of the data. The described PR-10 protein search database was used to identify peptides, while taking fixed (carbamidomethylation) and variable (oxidation of Methionine) modifications into account. After processing by PLGS, the so-called Exact Mass and Retention Time (EMRT) table was exported and reclustered using the PACP tool [59] to correct potential misalignments and split peak detection errors. Retention time was normalized and the reclustered EMRT table was further analyzed in Excel.

## Authors' contributions

MFS coordinated the study, performed the analysis and drafted the manuscript. WPCW performed the cloning and sequencing. HHGC performed the SDS-PAGE and Q-TOF LC- MSMS experiments, and participated in drafting the manuscript. AHPA participated in designing the study and in analyzing the Q-TOF LC- MS/MS data. LJWJG participated in the design and coordination of the study. MJMS participated in the design of the study and the analysis of the sequence data. All authors have read and approved the final manuscript.
